# Association of myocardial and liver T2* iron measurements with systolic and diastolic function by CMR feature tracking strain analysis

**DOI:** 10.3389/fcvm.2025.1547161

**Published:** 2025-03-18

**Authors:** Hugo G. Quezada-Pinedo, Benedikt Bernhard, Jan C. Zurkirchen, Anselm W. Stark, Noushin Sadat Ahanchi, Catherine Gebhard, Daniel Ott, Alan A. Peters, Hendrik von Tengg-Kobligk, Jonathan Schütze, Adam Bakula, Andreas Wahl, Kim N. Cajachagua-Torres, Taulant Muka, Christoph Gräni

**Affiliations:** ^1^ Department of Population Health Sciences, Duke University School of Medicine, Durham, NC, United States; ^2^ Department of Cardiology, Bern University Hospital, University of Bern, Bern, Switzerland; ^3^ Institute of Social and Preventive Medicine (ISPM), Graduate School of Health Sciences, University of Bern, Bern, Switzerland; ^4^ Graduate School for Health Sciences, University of Bern, Bern, Switzerland; ^5^ Department of Diagnostic, Interventional and Pediatric Radiology, Inselspital, Bern University Hospital, University of Bern, Bern, Switzerland; ^6^ Department of Pediatrics, New York University Grossman of Medicine, New York University, New York, NY, United States; ^7^ Epistudia, Bern, Switzerland

**Keywords:** T2*, iron overload, retrospective studies, feature tracking, strain, diastolic function

## Abstract

**Background/Objectives:**

Myocardial and liver iron overload can be assessed through T2* in magnetic resonance imaging (MRI). It is unclear, how T2* measurements are associated with systolic and diastolic left ventricular function assessed by novel feature tracking (FT) strain.

**Methods:**

Consecutive patients with suspected iron overload undergoing MRI T2* were retrospectively included. T2* was studied continuously and in categories: normal myocardial iron status (T2* ≥ 20 ms), myocardial iron overload (T2* < 20 ms), normal liver iron status (T2* ≥ 15.4 ms) and liver iron overload (T2* < 15.4 ms). Multivariable regression models were used to assess associations between T2* and FT strain.

**Results:**

Among 172 participants, longitudinal e/a ratio [−0.17 (−0.27, −0.08), *p* = 0.001], longitudinal early diastolic strain rate [−0.13 (−0.23, −0.03), *p* = 0.014], circumferential late diastolic strain rate [0.18 (0.03, 0.32), *p* = 0.016], longitudinal late diastolic strain rate [0.20 (0.03, 0.36), *p* = 0.019] were associated with higher T2*. Liver iron overload was associated with circumferential systolic strain rate [−0.42 (−0.74, −0.09), *p* = 0.014] and longitudinal early diastolic strain rate [0.27 (0.04, 0.49), *p* = 0.023]. Combined liver and myocardial iron overload were associated with longitudinal e/a ratio [0.72 (0.19, 1.24), *p* = 0.008]. No associations of T2* values with systolic function were found.

**Conclusion:**

Liver and a combination of myocardial and liver iron overload were associated with increased early diastolic filling and increased e/a ratio respectively, which may serve as markers of diastolic dysfunction. Impaired diastolic function, even in the absence of myocardial iron overload was associated with liver iron metabolism and may indicate early cardiac involvement, while left ventricular systolic function is still preserved.

## Introduction

Elevated myocardial iron levels can lead to oxidative stress, cellular damage, and impaired cardiac function (1). In recent years, significant advancements were made in the noninvasive assessment of myocardial and liver iron status using magnetic resonance imaging (MRI) (2, 3). This non-invasive technique allows not only to precisely measure myocardial and liver iron content, but also provides information on cardiac structure and function (4). Traditional serological measures of iron levels, such as ferritin, have limitations in accurately evaluating liver and myocardial iron content (5). Liver and cardiac MRI using T2* sequences offer a highly sensitive and specific approach to quantify tissue iron levels in a non-invasive manner (6). Novel cardiac MRI feature tracking (FT) strain analysis is able to quantify myocardial deformation in different orientations over an entire cardiac cycle, which allows for granular systolic and diastolic functional assessments. FT strain assessments demonstrated high reproducibility and were discussed to inherit prognostic value increment to that of left ventricular ejection fraction (LVEF) in various cardiac diseases (7). Limited data exists on whether systolic and diastolic myocardial function quantified through MRI strain analysis, are affected by myocardial iron deposition (8, 9). The hypothesis investigated in this retrospective cohort study was that changes in cardiac function quantified by MRI FT strain might be associated with myocardial and or liver iron status assessed by T2* analysis and could serve as an indicator of early cardiac involvement.

## Methods

### Study design

Consecutive patients with confirmed or suspected iron overload, who were referred for assessment of hepatic and cardiac MRI T2* at the University Hospital Bern, Switzerland were retrospectively included between 01/2010 and 12/2022. The study was approved by the Cantonal Ethics Committee (BASEC 2017-01112), and all participants gave written informed consent for the scientific use of their data.

### Cardiac magnetic resonance protocol

Scans were performed on a 1.5 Tesla MRI system (Magnetom Aera, Siemens Healthineers, Erlangen, Germany). Standard steady state-free precession 8 mm slice thickness cine images were acquired covering the left ventricle (LV) in three long axis views and a short-axis stack without interslice gap for the assessment of LV function and dimensions. Myocardial iron status was measured by the Siemens product provided T2* mapping sequence. Lower levels of T2* are indicative of higher stored iron levels. Iron status was studied as a continuous variable estimated by the T2* time and as categorial variable, using a cutoff of T2* ≥ 20 ms for normal myocardial iron status and <20 ms for myocardial iron overload. Liver iron status was classified as normal (T2* ≥ 15.4 ms) and liver iron overload (T2* < 15.4 ms) (10, 11). For further clinical interpretation, we classified iron status into: normal; isolated liver iron overload; and liver and myocardial iron overload.

### Assessment of myocardial function

**Figure 1:**
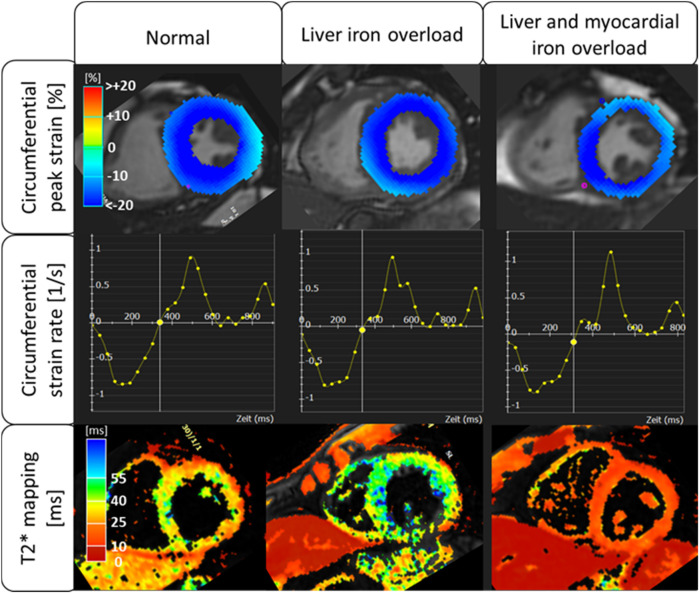
Selected strain analysis and T2* evaluation in patients with normal, liver iron overload, and liver and myocardial iron overload.

Cardiac dimensions, function and FT strain were analyzed on the commercially available software platform CVI42 (Circle Cardiovascular Imaging, Calgary, Canada) (Figure 1). In a first step, a short axis stack and three long axis (4-chamber, 3-chamber and 2-chamber) cine sequences were loaded into the software. Epi- and endocardial contours were automatically generated for end-diastole, checked by the reader for plausibility and adjusted if necessary. Papillary muscles were excluded from the LV myocardium. The software tracks contours through the full cardiac cycle to derive strain in various orientations, LV end-diastolic volume (LVEDV), LV end-systolic volume (LVESV), LV ejection fraction (LVEF), LV mass (LVM) and LV mass-to-volume ratio (LMVR), which serves as a marker of concentric remodeling (calculated as LVM/LVEDV). Strain analysis is performed in longitudinal (deformation from base to apex in the 2-, 3- and 4 chamber view), circumferential (deformation by change in the cardiac perimeter in the short axis stack) and radial (deformation by thickening of cardiac wall in the short axis stack) orientation (12). Diastolic function was assessed by selecting the early diastolic and late diastolic peaks, which depict left ventricular filling E and A waves, in strain rate and velocity in radial, circumferential and longitudinal orientation. Further, the E/A-ratio was calculated.

### Statistical analysis

Normality of continuous variables were evaluated by visual inspection of histograms and were presented as mean ± standard deviation (SD) if normally distributed or as median with 25th–75th percentile if non-normally distributed. Categorical variables are presented as absolute numbers (n) and percentages (%). Associations between myocardial iron status and FT strain parameters were examined using linear regression models considering myocardial iron status as an independent continuous or categorical variable. To facilitate comparisons of effect sizes, we calculated the standard deviation scores (SDS) [(observed value−mean)/SD] of the sample distribution for myocardial iron status and the cardiovascular measurements and FT strain parameters. Models were adjusted for cardiovascular risk factors i.e., smoking status, hypertension, diabetes, age and sex. We conducted various sensitivity analysis. First, we explored the association between liver and myocardial T2* with traditional measurements of cardiovascular structure and function (LVEF, LVEDV, LESV, LVM, LMVR). Second, to explore the potential influence of confounding by indication, we conducted a sensitivity analysis restricted to participants with MRI T2* normal levels. This approach was chosen because individuals with normal iron levels are less likely to receive MRI examination. Third**,** to explore if myocardial fibrosis influences our results, we additionally adjusted our models for the presence of late gadolinium enhancement (LGE). The BSA-adjusted SDS for these cardiovascular function and structure measurements was used in all our analysis. Statistical analyses were performed using R software version 4.3.2 (R Foundation, Vienna, Austria). As this study was designed as an exploratory analysis, we decided not to adjust for multiple testing.

## Results

### Participant characteristics

A total of 172 patients with a mean age of 46.8 ± 19.0 years, undergoing myocardial and liver T2* MRI were included. Clinical characteristics of participants are presented in Table 1. Among those, 54 (31.4%) were female, 14 (8.1%) were smokers, 21 (12.2%) had hypertension, 6 (3.5%) had diabetes, 69 (40.1%) were diagnosed with hemochromatosis, 41 (23.8%) had thalassemia, and 84 (48.8%) had elevated serum ferritin levels (>150 *μ*g/L for females or >300 *μ*g/L for males). Mean myocardial T2* was 34.7 ± 11.2 ms and the mean liver T2* was 14.1 ± 11.4 ms. Isolated liver iron overload as measured by T2* was found in 100 (58.1%) patients, while combined liver and myocardial iron overload was observed in 9 (5.2%) patients. No patients exhibited normal liver T2* with concurrent myocardial iron overload. Cardiac functional analysis revealed a mean left ventricular ejection fraction (LVEF) of 60.3 ± 9.4%, a global longitudinal peak strain (GLS) of −16.5 ± 3.3%, a global circumferential peak strain (GCS) of −16.8 ± 3.1%, and a global radial peak strain (GRS) of 26.9 ± 6.6% (Table 1).

**Table 1:** Participant's characteristics.

	*N*	Total	Normal	Liver iron overload only	Liver and myocardial iron overload	*p*-values normal vs. liver iron overload	*p*-values normal vs. liver and myocardial iron overload
		*n* = 172	*n* = 63	*n* = 100	*n* = 9		
Age (years)	172	46.8 (19.0)	52.2 (17.2)	45.1 (19.0)	27.2 (15.7)	**0**.**015**	**0**.**001**
Sex, female	172	54 (31.4)	18 (28.6)	31 (31.0)	5 (55.6)	0.878	0.214
Smoking, yes (%)	172	14 (8.1)	5 (7.9)	9 (9.0)	0 (0)	1.000	0.861
Hypertension, yes (%)	172	21 (12.2)	9 (14.3)	12 (12.0)	0 (0)	0.854	0.501
Diabetes, yes (%)	172	6 (3.5)	2 (3.2)	3 (3.0)	1 (11.1)	1.000	0.824
Hemochromatosis, yes (%)	172	69 (40.1)	32 (50.8)	35 (35.0)	2 (22.2)	0.067	0.212
Thalassemia, yes (%)	172	41 (23.8)	9 (14.3)	25 (25.0)	7 (77.8)	0.149	**<0**.**001**
High ferritin, yes (%)	172	84 (48.8)	31 (49.2)	51 (51.0)	2 (22.2)	0.950	0.245
Cardiac measurements							
Myocardial T2* (ms)	172	34.7 (11.2)	37.1 (12.7)	35.1 (8.4)	13.5 (4.6)	0.275	**<0**.**001**
Liver T2* (ms)	172	14.1 (11.4)	24.6 (12.4)	8.4 (4.2)	4.6 (2.2)	**<0**.**001**	**<0**.**001**
LVEF (%)	134	60.3 (9.4)	59.8 (9.6)	61.0 (9.2)	54.0 (9.8)	0.482	0.262
LVEDV (ml)	135	152.8 (56.9)	163.9 (62.4)	145.4 (51.6)	141.4 (62.7)	0.076	0.479
LVESV (ml)	135	63.5 (39.0)	69.4 (46.9)	58.8 (31.8)	68.6 (39.8)	0.152	0.968
LVM (g)	102	139.2 (107.6)	153.2 (143.5)	124.1 (40.8)	124.0 (25.5)	0.163	0.255
LMVR	102	0.9 (0.7)	0.9 (1.0)	0.8 (0.2)	0.8 (0.1)	0.267	0.369
Strain analysis							
GLS (%)	151	−16.5 (3.3)	−15.8 (3.7)	−16.9 (3.0)	−16.2 (3.0)	0.075	0.776
GCS (%)	152	−16.8 (3.1)	−16.4 (3.4)	−17.1 (2.8)	−16.4 (3.1)	0.246	0.964
GRS (%)	152	26.9 (6.6)	26.2 (7.2)	27.5 (6.2)	25.7 (6.3)	0.286	**0**.**839**
Longitudinal systolic strain rate	151	−0.8 (0.2)	−0.8 (0.2)	−0.9 (0.2)	−0.8 (0.2)	0.029	0.786
Circumferential systolic strain rate	152	−0.9 (0.2)	−0.9 (0.2)	−0.9 (0.2)	−0.9 (0.1)	0.007	0.554
Circumferential e/a ratio	163	2.2 (1.9)	1.8 (1.4)	2.3 (2.0)	3.8 (2.7)	0.052	0.054
Circumferential early diastolic strain rate (/s)	170	0.8 (0.3)	0.7 (0.3)	0.8 (0.3)	1.0 (0.3)	**0**.**007**	**0**.**014**
Circumferential late diastolic strain rate (/s)	164	0.5 (0.2)	0.5 (0.2)	0.5 (0.2)	0.3 (0.1)	0.290	**0**.**004**
Longitudinal e/a ratio	165	1.7 (1.3)	1.3 (0.7)	1.8 (1.3)	3.5 (2.4)	**0**.**001**	**0**.**030**
Longitudinal early diastolic strain rate (/s)	170	0.8 (0.4)	0.7 (0.4)	0.9 (0.3)	1.1 (0.4)	**0**.**002**	**0**.**011**
Longitudinal late diastolic strain rate (/s)	165	0.6 (0.2)	0.6 (0.3)	0.6 (0.2)	0.4 (0.2)	0.594	**0**.**015**

Values are means (SD), or valid percentages (absolute numbers). Myocardium iron status was classified as: normal (T2* ≥ 20 ms) and myocardium iron overload (T2* < 20 ms). Liver iron status was classified as normal (T2* ≥ 15.4 ms) and liver iron overload (T2* < 15.4 ms). LVEDV, Left ventricular end-diastolic volume; LVESV, left ventricular end-diastolic volume; LVEF, left ventricular ejection fraction, LVM, left ventricular mass; LMVR, left ventricular mass-to-volume ratio. GLS, global longitudinal peak strain; GCS, global circumferential peak strain; GRS, global radial peak strain. *P*-value for difference was calculated using Student's *t*-test for continuous normally distributed variables, Mann–Whitney *U* test for continuous not normally distributed variables, and chi-square test for categorical variables.

Bold values represent *p* < 0.05.

### Crude association of FT strain parameters with myocardial and liver iron status

Liver iron overload by T2*, as compared to normal hepatic iron levels, was associated with a significantly lower circumferential systolic strain rate [*β* = −0.56 (−0.93, −0.20) standard deviation score (SDS), *p* = 0.003], higher circumferential e/a ratio [0.33 (0.04, 0.63) SDS, *p* = 0.028], higher circumferential early diastolic strain rate [0.40 (0.12, 0.68) SDS, *p* = 0.006], lower longitudinal systolic strain rate [−0.41 (95% CI: −0.78, −0.05) SDS, *p* = 0.028], higher longitudinal e/a ratio [0.38 (0.12, 0.64) SDS, *p* = 0.004], and higher longitudinal early diastolic strain rate [0.44 (0.17, 0.70) SDS, *p* = 0.001]. In patients with combined liver and myocardial iron overload an even stronger association to higher circumferential early diastolic strain rate [0.87 (0.18, 1.55) SDS, *p* = 0.014], higher longitudinal e/a ratio [1.33 (0.71, 1.95) SDS, *p*-value = *p* < 0.001] and higher longitudinal early diastolic strain rate [0.97 (0.33, 1.60) SDS, *p* = 0.003] was observed. No other associations between hepatic and myocardial iron overload and cardiac measurements, were found (Supplementary Table S1).

When studying T2* continuously, similar associations were found and an increase in liver T2* by 1 SDS was associated with a lower circumferential e/a ratio [−0.15 (−0.28, −0.01), SDS, *p* = 0.037], lower circumferential early diastolic strain rate [−0.15 (−0.28, −0.02) SDS, *p* = 0.028], higher circumferential late diastolic strain rate [0.22 (0.06, 0.38), SDS, *p* = 0.008], lower longitudinal e/a ratio [−0.23 (−0.35, −0.10) SDS, *p* < 0.001], lower longitudinal early diastolic strain rate [−0.18 (−0.30, −0.05) SDS, *p* = 0.006], and higher longitudinal late diastolic strain rate [0.22 (0.06, 0.39) SDS, *p* = 0.009]. When myocardial T2* was analyzed as a continuous variable, no significant associations were observed between myocardial T2* levels and cardiac function (Supplementary Table S1).

### Adjusted associations of FT strain parameters with myocardial and liver iron status

**Figure 2:**
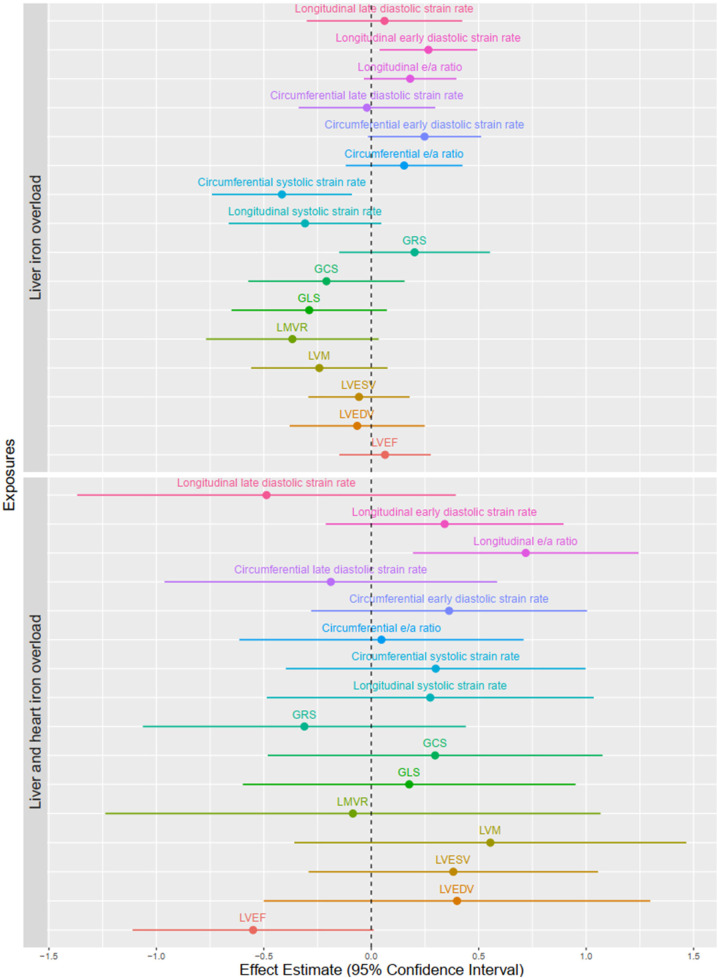
Associations of T2* liver and myocardial in categories and cardiac measurements. Values are linear regression betas (95% CI) and reflect the change in outcomes in SDS when a subject have liver or liver and myocardial iron overload as compared to the normal group. Models were adjusted for smoking status, hypertension, diabetes, age and sex. Myocardium iron status was classified as: normal (T2* ≥ 20 ms) and myocardium iron overload (T2* < 20 ms). Liver iron status was classified as normal (T2* ≥ 15.4 ms) and liver iron overload (T2* < 15.4 ms). LVEDV, left ventricular end-diastolic volume; LVESV, left ventricular end-diastolic volume; LVEF, left ventricular ejection fraction; LVM, left ventricular mass; LMVR, left ventricular mass-to-volume ratio. GLS, global longitudinal peak strain; GCS, global circumferential peak strain; GRS, global radial peak strain.

**Figure 3:**
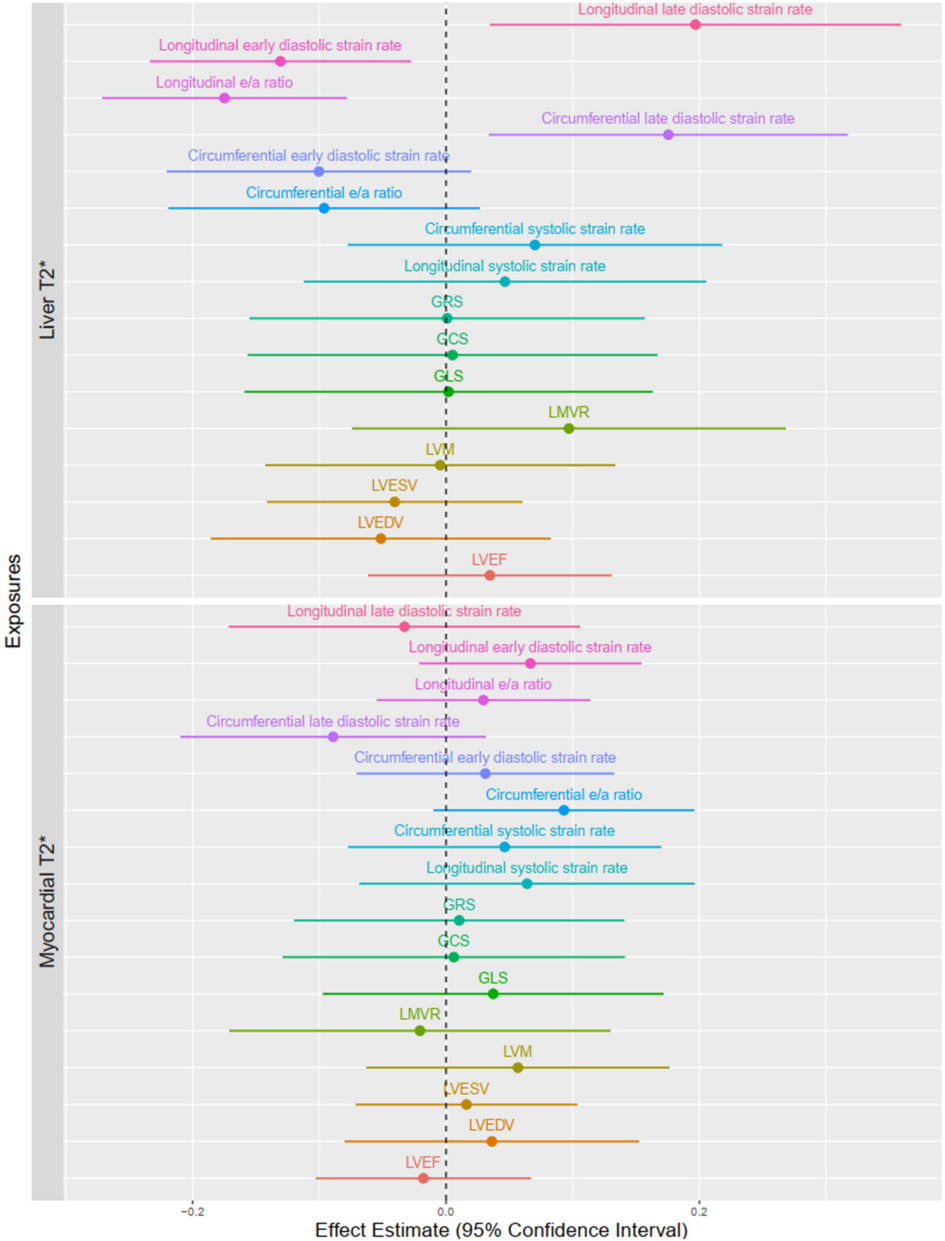
Associations of continuous T2* liver and myocardial and cardiac measurements. Circles are linear regression betas (95% CI) and reflect the change in outcomes in SDS per SDS change in iron status. Models were adjusted for smoking status, hypertension, diabetes, age and sex. LVEDV, left ventricular end-diastolic volume; LVESV, left ventricular end-diastolic volume; LVEF, left ventricular ejection fraction; LVM, left ventricular mass; LMVR, left ventricular mass-to-volume ratio. GLS, global longitudinal peak strain; GCS, global circumferential peak strain; GRS, global radial peak strain.

After adjustment for smoking status, hypertension, diabetes, age and sex, a decrease in circumferential systolic strain rate [*β* = −0.42 (95% CI: −0.74, −0.09) SDS, *p* = 0.014] and an increase in longitudinal early diastolic strain rate [0.27 (0.04, 0.49) SDS, *p* = 0.023] remained independently associated with liver iron overload. Combined liver and myocardial iron overload were independently associated with higher longitudinal e/a ratio [0.72 (0.19, 1.24) SDS, *p* = 0.008] (Figures 2,3, Supplementary Table S2).

After implementing T2* as a continuous variable into adjusted analysis, an increase of hepatic T2* by 1 SDS was associated with an increase in circumferential late diastolic strain rate [0.18 (0.03, 0.32) SDS; *p* = 0.016], a decrease of longitudinal e/a ratio [−0.17 (−0.27, −0.08) SDS, *p* = 0.001], a decrease of longitudinal early diastolic strain rate [−0.13 (−0.23, −0.03) SDS; *p* = 0.014], and an increase in longitudinal late diastolic strain rate [0.20 (0.03, 0.36) SDS; *p* = 0.019] (Figures 2,3, Supplementary Table S2). In a sensitivity analysis (Supplementary Table S3), considering only participants without iron overload, a higher liver T2* was associated higher circumferential late diastolic strain rate [0.31 (0.09, 0.53) SDS, *p* = 0.008], lower longitudinal e/a ratio (−0.14 (−0.24, −0.04) SDS, *p* = 0.01, higher longitudinal late diastolic strain rate [0.33 (0.01, 0.65) SDS, *p* = 0.045]. Moreover, higher myocardial T2* was associated with higher longitudinal e/a ratio [0.09 (0.01, 0.17) SDS, *p* = 0.030]. In another sensitivity analysis, additional adjustment for LGE presence showed that liver and heart iron overload compared to normal levels was associated with higher circumferential peak strain [3.17 (0.42, 5.91) SDS, *p* = 0.032] (Supplementary Table S4), lower LVEF [−1.91 (−3.39, −0.42) SDS, *p* = 0.017] and circumferential early diastolic strain rate [−1.27 (−2.46, −0.08) SDS, *p* = 0.046]. Similarly, when evaluated T2* continuously, higher myocardial T2* was associated with higher circumferential e/a ratio [0.19 (0.02, 0.37) SDS, *p* = 0.035], longitudinal e/a ratio (0.19 (0.04, 0.33 SDS, *p* = 0.015) and lower longitudinal late diastolic strain rate [−0.30 (−0.58, −0.02) SDS, *p* = 0.047] (Supplementary Table S4).

## Discussion

In this retrospective cohort study, we found that liver and the combination of liver and myocardial iron overload by MRI T2* were associated with lower early diastolic filling and increased e/a ratio, respectively, which may serve as markers of diastolic dysfunction. Our findings indicate that initial disturbances in liver iron metabolism predominantly impact diastolic function, while left ventricular systolic function is still preserved. Our study did not show any association between iron status and traditional cardiac metrics such as left ventricular ejection fraction, left ventricular end-diastolic volume, or left ventricular mass. This suggests that early cardiac changes might be more effectively identified using strain analysis, which could be incorporated into predictive models for early detection of cardiovascular alterations due to increased iron levels. After controlling for LGE, the T2* results showed ambiguous findings without a clear directional trend. This may be due to the limited statistical power, as LGE was only performed in a minority of patients. Therefore, the LGE adjusted results should be interpreted with caution.

Previous research indicates that individuals with increased iron levels in the liver and myocardium face a higher risk of cardiovascular morbidity and mortality. A study of 422 patients with hemochromatosis, demonstrated that iron overload multiplied the risk of death by five times in subjects whose ferritin levels were above 1,000 *µ*g/L (13). Similarly, in a previous Mendelian randomization study, genetically increased serum iron and transferrin saturation were associated with higher mortality risk (14). Adequate risk prediction and the early detection of cardiac involvement could help to tailor treatment to the individual risk of each patient.

Previous research indicated that the FT diastolic strain rate and the e/a ratio could adequately reflect diastolic function and are reproducible measurements (15, 16). In the present study, the analysis of iron status revealed an association between liver iron overload and diastolic strain. This aligns with the restrictive phenotype typically seen in iron overload cardiomyopathy, which is characterized by early diastolic dysfunction (17). Similarly, in our previous study, we identified that in boys, higher levels of hemoglobin during pregnancy were associated with lower right ventricular end-diastolic volume (RVEDV) and left ventricular end-diastolic volume (LVEDV) (18). Moreover, in the current study, the association between liver iron overload and impaired diastolic strain rate was independent from traditional measurements of cardiac function and structure (like LVEF, LVEDV, LESV, LVM, and LMVR), which showed no significant associations with iron status in the liver or myocardium. These observations suggest that subclinical abnormalities have already occurred in the myocardium and contribute to impaired diastolic function, even in instances where iron levels fall below the threshold for myocardial iron overload.

Previous studies also found an association of iron overload to systolic strain by either CMR or echocardiography (19–21). The patient population investigated in the present study was relatively healthy, young and frequently received treatment for iron overload, which might explain the normal systolic strain values. Supporting this, previous studies have shown that the heart can withstand oxidative stress from gradual iron accumulation for a prolonged period before systolic heart changes occur. If quantified by traditional markers of LV function, such as LVEF, systolic function and volumetric measurements are often normal despite substantial iron overload (20, 22). Currently, there is no available guideline for the treatment of patients with subclinical myocardial iron overload and it is unknown how this lack of standardized treatment influences cardiac dysfunction. Further research is needed to evaluate the long-term effects of early diastolic dysfunction in iron overload with regard to cardiovascular outcomes and response to therapy. It is possible that patients with normal iron levels have a milder disease or adequate treatment and were less likely to receive MRI T2* examination, potentially affecting our ability to identify associations. Our sensitivity analysis including only participants with normal MRI T2* levels showed that the identified association between liver T2* with circumferential late diastolic strain rate and longitudinal e/a ratio remained while the association between myocardial T2* and longitudinal e/a ratio become significant. These results showed consistent association with the total population and suggest that changes can be present even within the normal MRI T2* range. However, as changes in myocardial function were observed in patients with normal T2* values, it cannot be fully ruled out that these alterations may be influenced by factors such as hemodynamic changes (e.g., altered preload/afterload, microvascular dysfunction, or subtle myocardial edema) rather than early myocardial involvement. Alternatively, the current T2* thresholds may lack sufficient sensitivity, and emerging evidence suggests that combining T2* with T1 mapping or strain imaging could enhance the detection of early myocardial changes (23, 24).

### Methodological considerations and limitations

The strengths of our study lie in the comprehensive data collected on tissue iron status, assessed through MRI liver and myocardial T2*, as well as the meticulous evaluation of cardiovascular function using strain analysis including diastolic function. The novel aspect of this study lies in the evaluation of diastolic FT strain in the context of iron overload. Moreover, prior studies predominantly used correlation analyses. In our study, we controlled for confounding factors such as smoking status, hypertension, diabetes, age, and sex. Althougth we studied our research question in a relatively well-managed population, we could show that mainly liver iron overload was an early marker of impaired myocardial function.

Several limitations of our findings should be taken into account. The present study was performed at a single center and cross-sectional in nature, which restricted our ability to assess the long-term effects of iron status and the influence of fluctuations in iron status on cardiac function. Our sample was limited in size, which made it impossible to explore other factors such as the interaction effects of sex and iron in cardiac function and structure. Moreover, our capacity to reach statistical significance may be constrained by the reduced number of participants exhibiting myocardial iron overload. The small sample size in certain categories may lead to higher variance in coefficient estimates and wider confidence intervals. However, our results are biologically plausible, supported by previous studies. Additionally, the effect size showed a consistent association with similar magnitudes and direction in both crude and adjusted models. We cannot provide data on the specific treatments received by the patients. We therefore cannot exclude potential confounding of chelator therapy which might have led to a normalization of diastolic strain rates. Also, we lack of data on the numeric serum ferritin levels. However, prior research has found a significant correlation between serum ferritin levels and liver as well as myocardial T2* values (25–27). Residual confounding due to factors contributing to increased filling pressures, such as myocardial fibrosis, left atrial or mitral valve abnormalities cannot fully be ruled out. Additionally, the effects of dietary habits and iron supplementation were not systematically assessed in our study. We included a relatively healthy cohort of young subjects and therefore still believe that comorbidities affecting diastolic function do not relevantly impact our results. Additionally, studies using real-world data can be beneficial in balancing statistical rigor with practical feasibility to provide new hypothesis (28). Moreover, we followed a hypothesis-driven approach, with our analysis supported by our previous studies on iron status and cardiovascular function, as well as the current literature in the field. However, additional analyses, such as a more comprehensive evaluation of diastolic function in CMR and the assessment of FT strain in other cardiac regions, including left atrial (LA) strain, could provide valuable insights and should be explored in future studies (15, 29). Another limitation is the lack of adjustment for multiple testing in order to minimize the risk of Type I errors. However, considering our prespecified hypothesis and the exploratory nature of this study, we decided not to make such adjustments, as they could also increase the risk of Type II errors. While a longitudinal analysis of changes in iron status and their effects on repeated cardiac outcome measurements is an important research question, our study is limited by the number of individuals with these measurements in our sample. Additionally, the use of real-world data presents further challenges, as measurement times are not standardized across individuals adding more heterogeneity. Future studies might benefit from assessing longitudinal evaluation of MRI T2* and FT strain measurements. Consequently, our findings should be interpreted with caution, and further research is necessary to validate and expand upon our results.

## Conclusion

Higher liver and myocardial iron status were associated with impaired diastolic function, reflected by increased early diastolic filling and reduced late diastolic strain rates, while systolic function remained unaffected. Early changes in liver iron metabolism, even without myocardial iron overload, may contribute to myocardial dysfunction. Strain analysis in patients with elevated liver MRI T2* could be of additional value in assessing these subtle myocardial alterations. Further investigation into the underlying mechanisms and clinical implications is needed.

## Data Availability

The datasets presented in this article are not readily available because of privacy or ethical restrictions. The data are available upon reasonable request to the corresponding author. Requests to access the datasets should be directed to Christoph Gräni, christoph.graeni@insel.ch.
