# Supplementary material for: Association of myocardial and liver T2* iron measurements with systolic and diastolic function by CMR feature tracking strain analysis
Source: Front Cardiovasc Med. 2025 Mar 18;12:1547161. doi: 10.3389/fcvm.2025.1547161 (PMC11958997; doi:10.3389/fcvm.2025.1547161)
Supplement: Supplementary file 1 [file Datasheet1.docx]

**Supplementary material**

**Association of Myocardial and Liver T2* Iron Measurements with Systolic and Diastolic Function by CMR Feature Tracking Strain Analysis**

Hugo G Quezada-Pinedo, MD, PhD

Table S1. Crude associations of myocardial and liver MRI T2* and cardiac measurements

| **Exposure (SDS)** | **Outcome (SDS)** | **Beta 95% CI** | **P-value** |
| --- | --- | --- | --- |
| **Liver iron overload** | LVEF | 0.08 (-0.14, 0.30) | 0.480 |
| **(n= 100)** | LVEDV | -0.02 (-0.35, 0.30) | 0.900 |
| **vs. Normal** | LVESV | -0.04 (-0.29, 0.21) | 0.757 |
| **(n= 63)** | LVM | -0.18 (-0.51, 0.15) | 0.287 |
|  | LMVR | -0.21 (-0.61, 0.18) | 0.293 |
|  | Longitudinal peak strain | -0.35 (-0.73, 0.03) | 0.071 |
|  | Circumferential peak strain | -0.28 (-0.67, 0.10) | 0.152 |
|  | Radial peak strain | 0.26 (-0.11, 0.63) | 0.168 |
|  | Circumferential systolic strain rate | -0.56 (-0.93, -0.20) | **0.003** |
|  | Circumferential e/a ratio | 0.33 (0.04, 0.63) | **0.028** |
|  | Circumferential early diastolic strain rate | 0.40 (0.12, 0.68) | **0.006** |
|  | Circumferential late diastolic strain rate | -0.24 (-0.59, 0.11) | 0.177 |
|  | Longitudinal systolic strain rate | -0.41 (-0.78, -0.05) | **0.028** |
|  | Longitudinal e/a ratio | 0.38 (0.12, 0.64) | **0.004** |
|  | Longitudinal early diastolic strain rate | 0.44 (0.17, 0.70) | **0.001** |
|  | Longitudinal late diastolic strain rate | -0.09 (-0.45, 0.28) | 0.635 |
| **Liver and myocardial iron overload** | LVEF | -0.40 (-0.98, 0.19) | 0.185 |
| **(n= 9)** | LVEDV | 0.32 (-0.63, 1.26) | 0.515 |
| **vs. Normal** | LVESV | 0.28 (-0.44, 1.00) | 0.449 |
| **(n= 63)** | LVM | 0.23 (-0.74, 1.21) | 0.639 |
|  | LMVR | -0.20 (-1.37, 0.96) | 0.734 |
|  | Longitudinal peak strain | -0.11 (-0.89, 0.67) | 0.783 |
|  | Circumferential peak strain | -0.01 (-0.81, 0.79) | 0.976 |
|  | Radial peak strain | -0.04 (-0.81, 0.72) | 0.910 |
|  | Circumferential systolic strain rate | -0.24 (-0.99, 0.51) | 0.532 |
|  | Circumferential e/a ratio | 0.57 (-0.14, 1.27) | 0.119 |
|  | Circumferential early diastolic strain rate | 0.87 (0.18, 1.55) | **0.014** |
|  | Circumferential late diastolic strain rate | -0.72 (-1.56, 0.12) | 0.094 |
|  | Longitudinal systolic strain rate | -0.12 (-0.88, 0.64) | 0.763 |
|  | Longitudinal e/a ratio | 1.33 (0.71, 1.95) | **<0.001** |
|  | Longitudinal early diastolic strain rate | 0.97 (0.33, 1.60) | **0.003** |
|  | Longitudinal late diastolic strain rate | -0.82 (-1.7, 0.06) | 0.069 |
| **Liver T2*** | LVEF | 0.02 (-0.08, 0.12) | 0.746 |
| **(n=172)** | LVEDV | -0.05 (-0.19, 0.09) | 0.517 |
|  | LVESV | -0.03 (-0.14, 0.07) | 0.550 |
|  | LVM | -0.03 (-0.18, 0.11) | 0.673 |
|  | LMVR | 0.09 (-0.09, 0.26) | 0.334 |
|  | Longitudinal peak strain | 0.04 (-0.13, 0.21) | 0.677 |
|  | Circumferential peak strain | 0.06 (-0.12, 0.23) | 0.527 |
|  | Radial peak strain | -0.04 (-0.21, 0.12) | 0.601 |
|  | Circumferential systolic strain rate | 0.14 (-0.02, 0.31) | 0.094 |
|  | Circumferential e/a ratio | -0.15 (-0.28, -0.01) | **0.037** |
|  | Circumferential early diastolic strain rate | -0.15 (-0.28, -0.02) | **0.028** |
|  | Circumferential late diastolic strain rate | 0.22 (0.06, 0.38) | **0.008** |
|  | Longitudinal systolic strain rate | 0.09 (-0.07, 0.26) | 0.281 |
|  | Longitudinal e/a ratio | -0.23 (-0.35, -0.10) | **<0.001** |
|  | Longitudinal early diastolic strain rate | -0.18 (-0.30, -0.05) | **0.006** |
|  | Longitudinal late diastolic strain rate | 0.22 (0.06, 0.39) | **0.009** |
| **Myocardial T2*** | LVEF | -0.02 (-0.11, 0.07) | 0.648 |
| **(n=172)** | LVEDV | 0.06 (-0.06, 0.18) | 0.351 |
|  | LVESV | 0.03 (-0.06, 0.12) | 0.531 |
|  | LVM | 0.05 (-0.07, 0.18) | 0.398 |
|  | LMVR | -0.05 (-0.2, 0.10) | 0.490 |
|  | Longitudinal peak strain | 0.03 (-0.12, 0.17) | 0.713 |
|  | Circumferential peak strain | -0.01 (-0.15, 0.14) | 0.905 |
|  | Radial peak strain | 0.02 (-0.11, 0.16) | 0.728 |
|  | Circumferential systolic strain rate | 0.04 (-0.10, 0.18) | 0.548 |
|  | Circumferential e/a ratio | 0.10 (-0.01, 0.22) | 0.076 |
|  | Circumferential early diastolic strain rate | 0.04 (-0.07, 0.16) | 0.478 |
|  | Circumferential late diastolic strain rate | -0.10 (-0.24, 0.04) | 0.151 |
|  | Longitudinal systolic strain rate | 0.06 (-0.08, 0.20) | 0.402 |
|  | Longitudinal e/a ratio | 0.04 (-0.07, 0.14) | 0.483 |
|  | Longitudinal early diastolic strain rate | 0.07 (-0.04, 0.18) | 0.191 |
|  | Longitudinal late diastolic strain rate | -0.04 (-0.18, 0.10) | 0.604 |

Values are linear regression betas (95%CI) and reflect the change in outcomes in SDS per SDS change in iron status or reflect the change in outcomes in SDS when a subject have liver or liver and myocardial iron overload as compared to the normal group. Myocardium iron status was classified as: normal (T2* ≥ 20 ms) and myocardium iron overload (T2* < 20 ms). Liver iron status was classified as normal (T2* ≥ 15.4 ms) and liver iron overload (T2* < 15.4 ms). Left ventricular end-diastolic volume (LVEDV), left ventricular end-diastolic volume (LVESV), left ventricular ejection fraction (LVEF), left ventricular mass (LVM) and left ventricular mass-to-volume ratio (LMVR). Bold values represent p<0.05.

Table S2. Adjusted associations of myocardial and liver MRI T2* and cardiac measurements.

| **Exposure (SDS)** | **Outcome (SDS)** | **Beta 95% CI** | **P-value** |
| --- | --- | --- | --- |
| **Liver iron overload** | LVEF | 0.06 (-0.15, 0.28) | 0.556 |
| **(n= 100)** | LVEDV | -0.07 (-0.38, 0.25) | 0.684 |
| **vs. Normal** | LVESV | -0.06 (-0.29, 0.18) | 0.636 |
| **(n= 63)** | LVM | -0.24 (-0.56, 0.08) | 0.139 |
|  | LMVR | -0.37 (-0.77, 0.03) | 0.077 |
|  | Longitudinal peak strain | -0.29 (-0.65, 0.07) | 0.120 |
|  | Circumferential peak strain | -0.21 (-0.57, 0.16) | 0.263 |
|  | Radial peak strain | 0.20 (-0.15, 0.55) | 0.262 |
|  | Longitudinal systolic strain rate | -0.31 (-0.66, 0.05) | 0.091 |
|  | Circumferential systolic strain rate | -0.42 (-0.74, -0.09) | **0.014** |
|  | Circumferential e/a ratio | 0.15 (-0.12, 0.42) | 0.271 |
|  | Circumferential early diastolic strain rate | 0.25 (-0.02, 0.51) | 0.067 |
|  | Circumferential late diastolic strain rate | -0.02 (-0.34, 0.30) | 0.901 |
|  | Longitudinal e/a ratio | 0.18 (-0.03, 0.4) | 0.102 |
|  | Longitudinal early diastolic strain rate | 0.27 (0.04, 0.49) | **0.023** |
|  | Longitudinal late diastolic strain rate | 0.06 (-0.30, 0.42) | 0.739 |
| **Liver and myocardial iron overload** | LVEF | -0.55 (-1.11, 0.01) | 0.057 |
| **(n= 9)** | LVEDV | 0.40 (-0.50, 1.30) | 0.387 |
| **vs. Normal** | LVESV | 0.38 (-0.29, 1.06) | 0.269 |
| **(n= 63)** | LVM | 0.55 (-0.36, 1.47) | 0.237 |
|  | LMVR | -0.09 (-1.24, 1.07) | 0.885 |
|  | Longitudinal peak strain | 0.18 (-0.60, 0.95) | 0.656 |
|  | Circumferential peak strain | 0.30 (-0.48, 1.08) | 0.456 |
|  | Radial peak strain | -0.31 (-1.06, 0.44) | 0.418 |
|  | Longitudinal systolic strain rate | 0.27 (-0.49, 1.04) | 0.480 |
|  | Circumferential systolic strain rate | 0.30 (-0.40, 1.00) | 0.401 |
|  | Circumferential e/a ratio | 0.05 (-0.61, 0.71) | 0.889 |
|  | Circumferential early diastolic strain rate | 0.36 (-0.28, 1.00) | 0.270 |
|  | Circumferential late diastolic strain rate | -0.19 (-0.96, 0.59) | 0.634 |
|  | Longitudinal e/a ratio | 0.72 (0.19, 1.24) | **0.008** |
|  | Longitudinal early diastolic strain rate | 0.34 (-0.21, 0.90) | 0.228 |
|  | Longitudinal late diastolic strain rate | -0.49 (-1.37, 0.39) | 0.280 |
| **Liver T2*** | LVEF | 0.03 (-0.06, 0.13) | 0.482 |
| **(n=172)** | LVEDV | -0.05 (-0.19, 0.08) | 0.454 |
|  | LVESV | -0.04 (-0.14, 0.06) | 0.433 |
|  | LVM | 0.00 (-0.14, 0.13) | 0.949 |
|  | LMVR | 0.10 (-0.07, 0.27) | 0.269 |
|  | GLS | 0.00 (-0.16, 0.16) | 0.981 |
|  | GCS | 0.01 (-0.16, 0.17) | 0.950 |
|  | GRS | 0.00 (-0.16, 0.16) | 0.991 |
|  | Circumferential systolic strain rate | 0.07 (-0.08, 0.22) | 0.354 |
|  | Circumferential e/a ratio | -0.10 (-0.22, 0.03) | 0.127 |
|  | Circumferential early diastolic strain rate | -0.10 (-0.22, 0.02) | 0.104 |
|  | Circumferential late diastolic strain rate | 0.18 (0.03, 0.32) | **0.016** |
|  | Longitudinal systolic strain rate | 0.05 (-0.11, 0.21) | 0.567 |
|  | Longitudinal e/a ratio | -0.17 (-0.27, -0.08) | **0.001** |
|  | Longitudinal early diastolic strain rate | -0.13 (-0.23, -0.03) | **0.014** |
|  | Longitudinal late diastolic strain rate | 0.20 (0.03, 0.36) | **0.019** |
| **Myocardial T2*** | LVEF | -0.02 (-0.10, 0.07) | 0.680 |
| **(n=172)** | LVEDV | 0.04 (-0.08, 0.15) | 0.544 |
|  | LVESV | 0.02 (-0.07, 0.10) | 0.719 |
|  | LVM | 0.06 (-0.06, 0.18) | 0.355 |
|  | LMVR | -0.02 (-0.17, 0.13) | 0.789 |
|  | GLS | 0.04 (-0.10, 0.17) | 0.589 |
|  | GCS | 0.01 (-0.13, 0.14) | 0.930 |
|  | GRS | 0.01 (-0.12, 0.14) | 0.876 |
|  | Longitudinal systolic strain rate | 0.06 (-0.07, 0.20) | 0.346 |
|  | Circumferential systolic strain rate | 0.05 (-0.08, 0.17) | 0.465 |
|  | Circumferential e/a ratio | 0.09 (-0.01, 0.20) | 0.079 |
|  | Circumferential early diastolic strain rate | 0.03 (-0.07, 0.13) | 0.551 |
|  | Circumferential late diastolic strain rate | -0.09 (-0.21, 0.03) | 0.149 |
|  | Longitudinal e/a ratio | 0.03 (-0.05, 0.11) | 0.494 |
|  | Longitudinal early diastolic strain rate | 0.07 (-0.02, 0.15) | 0.139 |
|  | Longitudinal late diastolic strain rate | -0.03 (-0.17, 0.11) | 0.644 |

Values are linear regression betas (95%CI) and reflect the change in outcomes in SDS per SDS change in iron status or reflect the change in outcomes in SDS when a subject have liver or liver and myocardial iron overload as compared to the normal group. Models were adjusted for smoking status, hypertension, diabetes, age and sex. Myocardium iron status was classified as: normal (T2* ≥ 20 ms) and myocardium iron overload (T2* < 20 ms). Liver iron status was classified as normal (T2* ≥ 15.4 ms) and liver iron overload (T2* < 15.4 ms). Left ventricular end-diastolic volume (LVEDV), left ventricular end-diastolic volume (LVESV), left ventricular ejection fraction (LVEF), left ventricular mass (LVM) and left ventricular mass-to-volume ratio (LMVR). Bold values represent p<0.05.

Table S3. Adjusted associations of myocardial and liver MRI T2* and cardiac measurements in participants normal MRI T2*.

| **Exposure (SDS)** | **Outcome (SDS)** | **Beta 95% CI** | **P-value** |
| --- | --- | --- | --- |
| **Liver T2*** | LVEF | 0.12 (-0.02, 0.26) | 0.105 |
| **(n=63)** | LVEDV | -0.09 (-0.31, 0.13) | 0.428 |
|  | LVESV | -0.08 (-0.25, 0.08) | 0.320 |
|  | LVM | -0.06 (-0.28, 0.17) | 0.617 |
|  | LMVR | -0.06 (-0.38, 0.26) | 0.711 |
|  | Longitudinal peak strain | -0.22 (-0.48, 0.05) | 0.118 |
|  | Circumferential peak strain | -0.18 (-0.45, 0.08) | 0.181 |
|  | Radial peak strain | 0.18 (-0.08, 0.43) | 0.185 |
|  | Circumferential systolic strain rate | -0.17 (-0.43, 0.10) | 0.220 |
|  | Circumferential e/a ratio | -0.13 (-0.28, 0.02) | 0.089 |
|  | Circumferential early diastolic strain rate | -0.11 (-0.25, 0.03) | 0.136 |
|  | Circumferential late diastolic strain rate | 0.31 (0.09, 0.53) | **0.008** |
|  | Longitudinal systolic strain rate | -0.16 (-0.41, 0.09) | 0.223 |
|  | Longitudinal e/a ratio | -0.14 (-0.24, -0.04) | **0.010** |
|  | Longitudinal early diastolic strain rate | -0.11 (-0.33, 0.11) | 0.342 |
|  | Longitudinal late diastolic strain rate | 0.33 (0.01, 0.65) | **0.045** |
| **Myocardial T2*** | LVEF | -0.02 (-0.13, 0.09) | 0.710 |
| **(n=63)** | LVEDV | 0.05 (-0.11, 0.22) | 0.522 |
|  | LVESV | 0.03 (-0.1, 0.15) | 0.672 |
|  | LVM | 0.06 (-0.1, 0.23) | 0.457 |
|  | LMVR | -0.08 (-0.32, 0.17) | 0.543 |
|  | Longitudinal peak strain | 0.09 (-0.12, 0.29) | 0.413 |
|  | Circumferential peak strain | 0.03 (-0.17, 0.24) | 0.747 |
|  | Radial peak strain | -0.02 (-0.21, 0.18) | 0.879 |
|  | Circumferential systolic strain rate | 0.11 (-0.09, 0.31) | 0.290 |
|  | Circumferential e/a ratio | 0.03 (-0.08, 0.15) | 0.598 |
|  | Circumferential early diastolic strain rate | 0.02 (-0.09, 0.13) | 0.735 |
|  | Circumferential late diastolic strain rate | -0.07 (-0.24, 0.11) | 0.446 |
|  | Longitudinal systolic strain rate | 0.13 (-0.07, 0.32) | 0.207 |
|  | Longitudinal e/a ratio | 0.09 (0.01, 0.17) | **0.030** |
|  | Longitudinal early diastolic strain rate | 0.09 (-0.08, 0.26) | 0.296 |
|  | Longitudinal late diastolic strain rate | -0.09 (-0.34, 0.16) | 0.483 |

Values are linear regression betas (95%CI) and reflect the change in outcomes in SDS per SDS change in iron status. Models were adjusted for smoking status, hypertension, diabetes, age and sex. Left ventricular end-diastolic volume (LVEDV), left ventricular end-diastolic volume (LVESV), left ventricular ejection fraction (LVEF), left ventricular mass (LVM) and left ventricular mass-to-volume ratio (LMVR). Bold values represent p<0.05.

Table S4. Adjusted associations of myocardial and liver MRI T2* and cardiac measurements (Additionally adjusted for late gadolinium enhancement).

| **Exposure (SDS)** | **Outcome (SDS)** | **Beta 95% CI** | **P-value** |
| --- | --- | --- | --- |
| **Liver iron overload** | LVEF | -0.41 (-0.91, 0.10) | 0.126 |
| **(n= 13)** | LVEDV | 0.05 (-0.69, 0.79) | 0.904 |
| **vs. Normal** | LVESV | 0.13 (-0.45, 0.71) | 0.675 |
| **(n= 23)** | LVM | -0.20 (-0.92, 0.53) | 0.597 |
|  | LMVR | -0.07 (-0.24, 0.11) | 0.455 |
|  | Longitudinal peak strain | 0.62 (-0.25, 1.48) | 0.173 |
|  | Circumferential peak strain | 0.80 (-0.16, 1.76) | 0.115 |
|  | Radial peak strain | -0.68 (-1.59, 0.23) | 0.153 |
|  | Circumferential systolic strain rate | -0.02 (-0.88, 0.84) | 0.962 |
|  | Circumferential e/a ratio | 0.06 (-0.53, 0.64) | 0.854 |
|  | Circumferential early diastolic strain rate | -0.02 (-0.48, 0.43) | 0.926 |
|  | Circumferential late diastolic strain rate | -0.45 (-1.43, 0.53) | 0.379 |
|  | Longitudinal systolic strain rate | 0.37 (-0.54, 1.28) | 0.435 |
|  | Longitudinal e/a ratio | 0.37 (-0.09, 0.83) | 0.127 |
|  | Longitudinal early diastolic strain rate | -0.08 (-0.46, 0.30) | 0.670 |
|  | Longitudinal late diastolic strain rate | -0.78 (-1.70, 0.15) | 0.110 |
| **Liver and heart iron overload** | LVEF | -1.91 (-3.39, -0.42) | **0.017** |
| **(n= 1)** | LVEDV | 0.36 (-1.78, 2.50) | 0.746 |
| **vs. Normal** | LVESV | 0.89 (-0.79, 2.56) | 0.308 |
| **(n= 23)** | LVM | 0.01 (-2.11, 2.12) | 0.994 |
|  | LMVR | -0.14 (-0.64, 0.37) | 0.598 |
|  | Longitudinal peak strain | 2.04 (-0.43, 4.50) | 0.116 |
|  | Circumferential peak strain | 3.17 (0.42, 5.91) | **0.032** |
|  | Radial peak strain | -2.68 (-5.26, -0.09) | 0.052 |
|  | Circumferential systolic strain rate | 1.92 (-0.52, 4.36) | 0.134 |
|  | Circumferential e/a ratio | -0.79 (-2.32, 0.74) | 0.320 |
|  | Circumferential early diastolic strain rate | -1.27 (-2.46, -0.08) | **0.046** |
|  | Circumferential late diastolic strain rate | -0.57 (-3.14, 2.01) | 0.669 |
|  | Longitudinal systolic strain rate | 1.81 (-0.79, 4.41) | 0.183 |
|  | Longitudinal e/a ratio | -0.71 (-1.93, 0.51) | 0.265 |
|  | Longitudinal early diastolic strain rate | -0.94 (-1.94, 0.05) | 0.074 |
|  | Longitudinal late diastolic strain rate | -0.49 (-2.91, 1.94) | 0.698 |
| **Liver T2*** | LVEF | 0.22 (-0.09, 0.53) | 0.174 |
| **(n= 37)** | LVEDV | -0.05 (-0.47, 0.36) | 0.809 |
|  | LVESV | -0.10 (-0.43, 0.22) | 0.541 |
|  | LVM | 0 (-0.41, 0.41) | 0.996 |
|  | LMVR | 0.03 (-0.07, 0.13) | 0.537 |
|  | Longitudinal peak strain | -0.38 (-0.89, 0.12) | 0.147 |
|  | Circumferential peak strain | -0.45 (-1.03, 0.14) | 0.147 |
|  | Radial peak strain | 0.37 (-0.17, 0.92) | 0.191 |
|  | Circumferential systolic strain rate | 0 (-0.50, 0.51) | 0.988 |
|  | Circumferential e/a ratio | 0.05 (-0.27, 0.37) | 0.766 |
|  | Circumferential early diastolic strain rate | 0.08 (-0.18, 0.34) | 0.558 |
|  | Circumferential late diastolic strain rate | 0.26 (-0.28, 0.79) | 0.354 |
|  | Longitudinal systolic strain rate | -0.22 (-0.75, 0.32) | 0.431 |
|  | Longitudinal e/a ratio | -0.14 (-0.41, 0.12) | 0.293 |
|  | Longitudinal early diastolic strain rate | 0.10 (-0.11, 0.32) | 0.349 |
|  | Longitudinal late diastolic strain rate | 0.37 (-0.14, 0.88) | 0.163 |
| **Myocardial T2*** | LVEF | -0.02 (-0.22, 0.17) | 0.822 |
| **(n = 37)** | LVEDV | -0.01 (-0.27, 0.24) | 0.921 |
|  | LVESV | 0 (-0.21, 0.20) | 0.963 |
|  | LVM | 0.06 (-0.19, 0.31) | 0.655 |
|  | LMVR | 0.03 (-0.03, 0.09) | 0.347 |
|  | Longitudinal peak strain | 0.19 (-0.11, 0.49) | 0.233 |
|  | Circumferential peak strain | 0.11 (-0.25, 0.47) | 0.561 |
|  | Radial peak strain | -0.11 (-0.45, 0.22) | 0.507 |
|  | Circumferential systolic strain rate | 0.19 (-0.11, 0.48) | 0.220 |
|  | Circumferential e/a ratio | 0.19 (0.02, 0.37) | **0.035** |
|  | Circumferential early diastolic strain rate | 0.01 (-0.14, 0.17) | 0.862 |
|  | Circumferential late diastolic strain rate | -0.25 (-0.55, 0.04) | 0.104 |
|  | Longitudinal systolic strain rate | 0.28 (-0.02, 0.59) | 0.077 |
|  | Longitudinal e/a ratio | 0.19 (0.04, 0.33) | **0.015** |
|  | Longitudinal early diastolic strain rate | 0 (-0.13, 0.12) | 0.990 |
|  | Longitudinal late diastolic strain rate | -0.30 (-0.58, -0.02) | **0.047** |

Values are linear regression betas (95%CI) and reflect the change in outcomes in SDS per SDS change in iron status or reflect the change in outcomes in SDS when a subject have liver or liver and myocardial iron overload as compared to the normal group. Models were adjusted for smoking status, hypertension, diabetes, age, sex and late gadolinium enhancement. Myocardium iron status was classified as: normal (T2* ≥ 20 ms) and myocardium iron overload (T2* < 20 ms). Liver iron status was classified as normal (T2* ≥ 15.4 ms) and liver iron overload (T2* < 15.4 ms). Left ventricular end-diastolic volume (LVEDV), left ventricular end-diastolic volume (LVESV), left ventricular ejection fraction (LVEF), left ventricular mass (LVM) and left ventricular mass-to-volume ratio (LMVR). Bold values represent p<0.05.
